# Optimal Treatment Selection in Sequential Systemic and Locoregional Therapy of Oropharyngeal Squamous Carcinomas: Deep Q-Learning With a Patient-Physician Digital Twin Dyad

**DOI:** 10.2196/29455

**Published:** 2022-04-20

**Authors:** Elisa Tardini, Xinhua Zhang, Guadalupe Canahuate, Andrew Wentzel, Abdallah S R Mohamed, Lisanne Van Dijk, Clifton D Fuller, G Elisabeta Marai

**Affiliations:** 1 Department of Computer Science University of Illinois at Chicago Chicago, IL United States; 2 Dipartimento di Elettronica Informazione e Bioingegneria Politecnico di Milano Milan Italy; 3 Department of Electrical and Computer Engineering University Of Iowa Iowa City, IA United States; 4 MD Anderson Cancer Center Houston, TX United States; 5 Department of Radiation Oncology The University of Texas Austin, TX United States

**Keywords:** digital twin dyad, reinforcement learning, head and neck cancer

## Abstract

**Background:**

Currently, selection of patients for sequential versus concurrent chemotherapy and radiation regimens lacks evidentiary support and it is based on locally optimal decisions for each step.

**Objective:**

We aim to optimize the multistep treatment of patients with head and neck cancer and predict multiple patient survival and toxicity outcomes, and we develop, apply, and evaluate a first application of deep Q-learning (DQL) and simulation to this problem.

**Methods:**

The treatment decision DQL digital twin and the patient’s digital twin were created, trained, and evaluated on a data set of 536 patients with oropharyngeal squamous cell carcinoma with the goal of, respectively, determining the optimal treatment decisions with respect to survival and toxicity metrics and predicting the outcomes of the optimal treatment on the patient. Of the data set of 536 patients, the models were trained on a subset of 402 (75%) patients (split randomly) and evaluated on a separate set of 134 (25%) patients. Training and evaluation of the digital twin dyad was completed in August 2020. The data set includes 3-step sequential treatment decisions and complete relevant history of the patient cohort treated at MD Anderson Cancer Center between 2005 and 2013, with radiomics analysis performed for the segmented primary tumor volumes.

**Results:**

On the test set, we found mean 87.35% (SD 11.15%) and median 90.85% (IQR 13.56%) accuracies in treatment outcome prediction, matching the clinicians’ outcomes and improving the (predicted) survival rate by +3.73% (95% CI –0.75% to 8.96%) and the dysphagia rate by +0.75% (95% CI –4.48% to 6.72%) when following DQL treatment decisions.

**Conclusions:**

Given the prediction accuracy and predicted improvement regarding the medically relevant outcomes yielded by this approach, this digital twin dyad of the patient-physician dynamic treatment problem has the potential of aiding physicians in determining the optimal course of treatment and in assessing its outcomes.

## Introduction

### Background

Head and neck cancer, which includes cancers of the larynx, throat, lips, mouth, nose, and salivary glands, is now an epidemic, with 65,000 new cases in the United States annually [1], whose treatment is, as in many other types of cancers, a dynamic and complex process. This therapy process involves making multiple, patient-specific treatment decisions to maximize efficacy—for example, reduction in tumor size, time of local region control, and survival time—while minimizing side effects [2-4]. For example, a specific patient may undergo radiotherapy (RT) alone, RT with concurrent chemotherapy (CC), or induction chemotherapy (IC) [5]. After each round of IC, a decision must be made whether to continue IC or start either RT or CC. These decisions are currently taken by clinicians or multidisciplinary tumor boards based on pretherapy patient characteristics or crude heuristics. Notably, current risk-prediction models (eg, American Joint Committee on Cancer [AJCC] staging) incorporated in clinical decision support systems do not by themselves systematically direct clinicians to select an appropriate treatment that incorporates *both* oncologic and toxicity end points.

**Figure 1:**
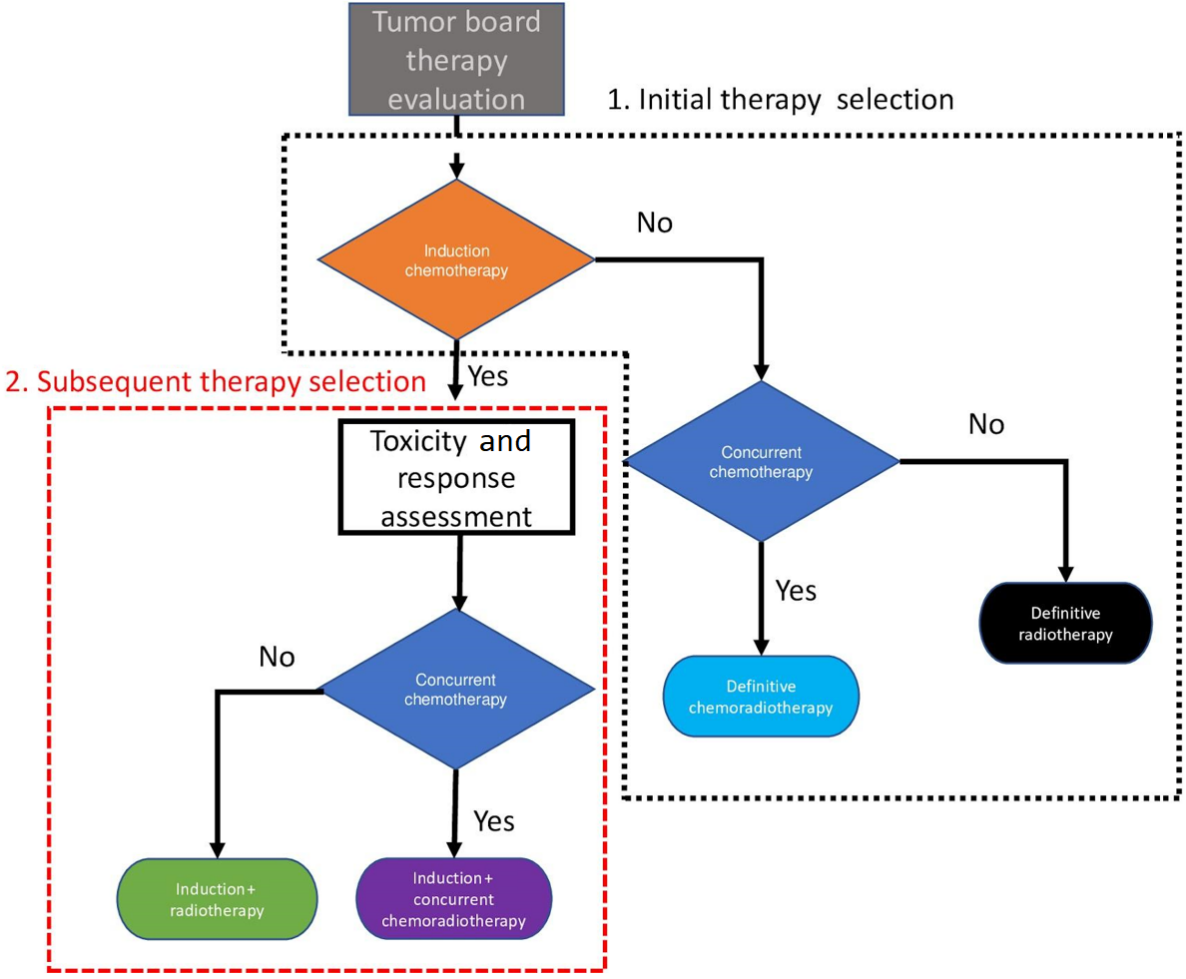
Overview of the therapy selection process, which shows two distinct phases: initial therapeutic selection and subsequent therapeutic selection.

Furthermore, disposition to initial IC is then followed by a second responsive disposition to either RT or concurrent chemoradiotherapy. Inferring the optimal treatment policies for multistage decisions (eg, which treatment to administer initially and then after observing treatment response; Figure 1) post hoc is challenging because an optimal therapy sequence cannot be readily *pieced together* from several single-stage decisions.

For this reason, in the absence of rigorous clinical trials comparing adaptive IC permutations with concurrent RT, group comparison is exceedingly difficult because simple models that account for confounders at initial disposition (eg, propensity scores) are unequipped to incorporate sequential decision processes (eg, the choice of CC *after* IC).

### Digital Twinning

To address multistage models of therapy selection that incorporate both relevant cancer and side-effect considerations, we introduce an approach based on *digital twinning*, a new concept adapted to health research from the industrial world, where a digital replica (*digital twin*) of a physical entity or process is virtually recreated, with similar elements and dynamics, to perform real-time optimization and testing [6]. In health care, coupled digital twins, that is, *digital twin dyads*, could be created for both patients and for the therapy process and used to inform in a quantitative manner adaptive therapy decision-making and allow personalization and optimization of health outcomes, prediction and prevention of adverse events, and planning interventions [7]. By leveraging a large number of head and neck cancer cases collected at a single institutional head and neck data tumor board at the MD Anderson Cancer Center (MDACC), we propose a methodology approach to leverage deep Q-learning (DQL) as a method to construct a *digital twin dyad* for simulation of therapy outcomes and potential implementation as a clinical decision aid. Q-learning is a recently developed machine learning method for supervised variable selection and weighting accounting for iterative processes [8].

In this paper, we apply for the first time Q-learning methodology to dynamically select treatment based on multiple clinically relevant outcomes from data specific to patients with head and neck cancer. We use these methods to construct and develop optimal dynamic treatment strategies, that is, digital twins of the therapy process. In conjunction with simulation models of patient data, the treatment prescription models form a patient-physician (prescriber) *digital twin dyad* in which the treatment prescription models act as a physician avatar by identifying the optimal treatment for the patient, whereas the simulation models represent the patient by predicting the outcome of the treatment sequence. We evaluate the results of this digital twin dyad approach on a curated data set of patients with head and neck cancer.

## Methods

### Overview

A state-of-the-art machine learning method applicable to the optimal therapy process problem is reinforcement learning (RL), in particular DQL [8-12]. DQL aims to solve problems in which a model has to choose among a series of options to maximize a certain goal in the given situation: the model observes a set of actions and the outcome these actions have, thus learning which choices are optimal and which are not. Q-learning is thus a type of machine learning that enables systems to automatically learn and improve from experience without being explicitly programmed. Q-learning has been shown to lead to valid results in a variety of medical problems, including the definition of a sequential multiple-assignment randomized trial [8,9], the optimal treatment of depression [8] and Attention-Deficit/Hyperactivity Disorder [9], and the breastfeeding habits that maximize child vocabulary development [10].

We used DQL to find a treatment policy that maximizes a linear combination of multiple patient outcomes; for example, toxicological and survival outcomes. We considered a 3-step Markov decision process (MDP), with 3 actions in each episode corresponding to the three treatment decision points for each patient:

1. Decision 1 (D1): *IC or not*
2. Decision 2 (D2): *CC or RT alone*
3. Decision 3 (D3): *neck dissection (ND) or not*

More details on the setup of the MDP are described in the following sections, including the reward functions and state variables.

### Patient Data Set

We performed a retrospective review of 536 patients with oropharyngeal squamous cell carcinoma who were treated at the MDACC between 2005 and 2013 (Tables 1-3). Radiomics analysis was performed [13,14] for the segmented [15,16] primary tumor volumes. Only patients with a minimum follow-up of 4 years or who died within 4 years were included in the data set. The 536 examples were partitioned into 2 distinct sets for training and testing using a 75% (n=402)-25% (n=134) random split. To save space, in Table 1, the results of all binary features are shown only for 1 outcome; the others can be derived directly by subtracting from 100%. For example, the figures for sex being female are 65 (12.1%), 47 (11.7%), and 18 (13.4%), under the respective column headings.

**Table 1:** Demographics of pretreatment features (before decision 1 [D1]: induction chemotherapy or not; N=536).

Characteristics				All patients (N=536)		Training set (n=402)		Testing set (n=134)
**Group 1: Pretreatment features (before D1)**								
	Age (years) at diagnosis, mean (SD)			58.9 (9.5)		58.5 (9.4)		60.2 (9.6)
	**Pathological grade, n (%)**							
		I	6 (1.1)		2 (0.5)		4 (3)	
		II	154 (28.7)		114 (28.4)		40 (29.9)	
		III	274 (51.1)		206 (51.2)		88 (65.7)	
		IV	3 (0.6)		1 (0.2)		2 (1.5)	
		Not available	99 (18.5)		79 (19.7)		20 (14.9)	
	**Sex (male), n (%)**			471 (87.9)		355 (88.3)		116 (86.6)
	**HPV^a^ or P16^b^ status, n (%)**							
		Negative	43 (8)		33 (8.2)		10 (7.5)	
		Positive	305 (56.9)		228 (56.7)		77 (57.5)	
		Unknown	188 (35.1)		141 (35.1)		47 (35.1)	
	**T^c^ category, n (%)**							
		T1	113 (21.1)		87 (21.6)		26 (19.4)	
		T2	219 (40.9)		156 (38.8)		63 (47)	
		T3	116 (21.6)		91 (22.6)		25 (18.7)	
		T4	86 (16)		67 (16.7)		19 (14.2)	
		Tx^d^	2 (0.4)		1 (0.2)		1 (0.7)	
	**N^e^ category (8th edition^f^), n (%)**							
		N0^g^	20 (3.7)		14 (3.5)		6 (4.5)	
		N1	249 (46.5)		181 (45)		68 (50.7)	
		N2	250 (46.6)		194 (48.3)		56 (41.8)	
		N3	17 (3.2)		13 (3.2)		4 (3)	
	**AJCC^h^ (8th edition), n (%)**							
		I	186 (34.7)		137 (34.1)		49 (36.6)	
		II	81 (15.1)		63 (15.7)		18 (13.4)	
		III	64 (11.9)		44 (10.9)		20 (14.9)	
		IV	203 (37.9)		157 (39.1)		46 (34.3)	
		Not available	2 (0.3)		1 (0.2)		1 (0.7)	
	**Smoking status at diagnosis, n (%)**							
		Current	115 (21.5)		85 (21.1)		30 (22.4)	
		Former	203 (37.9)		151 (37.6)		52 (38.8)	
		Never	218 (40.7)		166 (41.3)		52 (38.8)	
	**Smoking status**							
		Packs per year, mean (SD)	17.7 (23.7)		16.7 (22.9)		20.5 (26)	
		Not available, n (%)	28 (4.7)		21 (5.2)		7 (5.2)	
	Aspiration rate before therapy (no), n (%)			16 (3)		14 (3.5)		2 (1.5)
	Number of affected lymph nodes, mean (SD)			2.0 (1.3)		2.1 (1.3)		1.8 (1)
	**Tumor laterality, n (%)**							
		Bilateral	21 (3.9)		16 (4)		5 (3.7)	
		Left	242 (45.1)		188 (46.8)		54 (40.3)	
		Right	273 (50.9)		198 (49.3)		75 (56)	
	**Tumor subsite, n (%)**							
		Base of tongue	266 (49.6)		204 (50.7)		62 (46.3)	
		Tonsil	223 (41.6)		158 (39.3)		65 (48.5)	
		Other	47 (8.8)		40 (10)		7 (5.2)	
	**Race, n (%)**							
		African American or Black	16 (3)		10 (2.5)		6 (4.5)	
		Asian	4 (0.7)		3 (0.7)		1 (0.7)	
		Hispanic or Latino	21 (3.9)		17 (4.2)		4 (3)	
		Native American	1 (0.2)		1 (0.2)		0 (0)	
		White or other	494 (92.2)		371 (92.3)		123 (91.8)	

^a^HPV: human papillomavirus.

^b^P16: protein expression 16.

^c^T: primary tumor.

^d^Tx: no information about the primary tumor or it cannot be measured.

^e^N: lymph nodes.

^f^American Joint Committee on Cancer’s Cancer Staging Manual, 8th edition.

^g^N0: nearby lymph nodes do not contain cancer.

^h^AJCC: American Joint Committee on Cancer.

**Table 2:** Feature demographics before and after decision junctions (N=536).

Characteristics				All patients (N=536), n (%)		Training set (n=402), n (%)		Testing set (n=134), n (%)
**Group 2: Post–** **induction chemotherapy–** **decision features (after D1^a^ and before D2^b^)**								
	**Prescribed chemotherapy**							
		None	342 (63.8)		250 (62.2)		92 (68.7)	
		Doublet	41 (7.6)		32 (8)		9 (6.7)	
		Triplet	143 (26.7)		111 (27.6)		32 (23.9)	
		Quadruplet	7 (1.3)		7 (1.7)		0 (0)	
		Not otherwise specified	3 (0.6)		2 (0.5)		1 (0.7)	
	Chemotherapy modification		85 (15.9)		65 (16.2)		20 (14.9)	
	**Chemotherapy modification type**							
		No dose adjustment	451 (84.1)		336 (83.6)		115 (85.8)	
		Dose modified	21 (3.9)		16 (4)		5 (3.7)	
		Dose delayed	10 (1.9)		9 (2.2)		1 (0.7)	
		Dose cancelled	18 (3.4)		13 (3.2)		5 (3.7)	
		Dose delayed and modified	6 (1.1)		5 (1.2)		1 (0.7)	
		Regimen modification	29 (5.4)		22 (5.5)		7 (5.2)	
		Unknown	1 (0.2)		1 (0.2)		0 (0)	
	Dose-limiting toxicity		95 (17.7)		73 (18.2)		22 (16.4)	
	**Dose-limiting toxicity g rade (also included for dermatological, neurological, gastrointestinal, hematological, nephrological, vascular, and infection [pneumonia])**							
		0	446 (83.2)		334 (83.1)		112 (83.6)	
		1	7 (1.3)		6 (1.5)		1 (0.7)	
		2	33 (6.2)		26 (6.5)		7 (5.2)	
		3	41 (7.6)		29 (7.2)		12 (9)	
		4	9 (1.7)		7 (1.7)		2 (1.5)	
	Imaging (yes)		194 (36.2)		152 (37.8)		42 (31.3)	
	Complete response, primary (1^c^, as opposed to 0^d^)		84 (15.7)		67 (16.7)		17 (12.7)	
	Complete response, nodal (1)		16 (3)		14 (3.5)		2 (1.5)	
	Parietal response, primary (1)		89 (16.6)		70 (17.4)		19 (14.2)	
	Parietal response, nodal (1)		156 (29.1)		125 (31.1)		31 (23.1)	
	Stable disease, primary (1)		11 (2.1)		8 (2)		3 (2.2)	
	Stable disease, nodal (1)		10 (1.9)		6 (1.5)		4 (3)	
**Group 3: Post–concurrent** **chemotherapy** **–decision features (after D2 and before D3^e^)**								
	**Concurrent chemotherapy regimen**							
		None	126 (23.5)		89 (22.1)		37 (27.6)	
		Platinum based	257 (47.9)		198 (49.3)		59 (44)	
		Cetuximab based	129 (24.1)		95 (23.6)		34 (25.4)	
		Other	24 (4.5)		20 (5)		4 (3)	
	Concurrent chemotherapy modification (1)		99 (18.5)		77 (19.2)		22 (16.4)	
	Complete response, primary 2 (1)		450 (84.1)		336 (83.8)		114 (85.1)	
	Complete response, nodal 2 (1)		247 (46.1)		186 (46.3)		61 (45.5)	
	Parietal response, primary 2 (1)		77 (14.4)		58 (14.4)		19 (14.2)	
	Parietal response, nodal 2 (1)		257 (47.9)		191 (47.5)		66 (49.3)	
	Stable disease, primary 2 (1)		2 (0.4)		2 (0.5)		0 (0)	
	Stable disease, nodal 2 (1)		10 (1.9)		6 (1.5)		4 (3)	
	Dose-limiting toxicity 2 (also included for dermatological, neurological, gastrointestinal, hematological, nephrological, vascular, and other)		102 (19)		80 (19.9)		22 (16.4)	
**Group 4: Primary outcomes after D3**								
	Four-year overall survival (alive)		457 (85.3)		344 (85.6)		113 (84.3)	
	Feeding tube 6 months (yes)		98 (18.3)		77 (19.2)		21 (15.7)	
	Aspiration rate after therapy (yes)		98 (18.3)		79 (19.7)		19 (14.2)	
	Dysphagia (yes)		154 (28.7)		122 (30.3)		32 (23.9)	

^a^D1: decision 1 (induction chemotherapy or not).

^b^D2: decision 2 (concurrent chemotherapy or radiotherapy alone).

^c^The patient survived for at least four years after the treatment ended.

^d^All other events.

^e^D3: decision 3 (neck dissection or not).

**Table 3:** Demographics of physicians’ decisions (N=536).

Characteristics			All patients (N=536), n (%)		Training set (n=402), n (%)		Testing set (n=134), n (%)
**Treatment decisions (made by physicians)**							
	D1^a^: yes	194 (36.2)		152 (37.8)		42 (31.3)	
	D2^b^: yes	410 (76.5)		313 (77.9)		97 (72.4)	
	D3^c^: yes	111 (20.7)		84 (20.9)		27 (20.1)	

^a^D1: decision 1 (induction chemotherapy or not).

^b^D2: decision 2 (concurrent chemotherapy).

^c^D3: decision 3 (neck dissection or not).

### Ethics Approval

The data were collected after approval from the MDACC institutional review board (PA16-0303 and retrospective RCR03-0800).

### Modeling

We focused on two outcome measures: (1) four-year *overall survival* (OS) as a single binary dichotomized outcome measure (ie, the patient survived for at least four years after the treatment ended, coded as 1, with all other events coded 0) and (2) the combination of *OS* and *dysphagia* (DP) as a multi-outcome measure. *DP* is defined as either *feeding-tube dependence* (FT) or *aspiration rate* (AR) 6 months after the end of treatment [17,18]. Note that although OS is encoded as a binary value, the outcome of treatment depends on the external situation and the treatment sequence applied; that is, both OS and DP are influenced by the treatment sequence applied. As a result, the whole problem is not a simple regression but bona fide RL with unknown transition probability. The combined outcome measure was computed only at the final step using the following formula:

OS–(FT+AR After Therapy–AR Before Therapy) **(1)**

Equation (1) was used as the total reward in training the DQL models*.* For each of these scenarios, the models were trained with and without the inclusion of radiomics features [19,20]. The reward is 0 for D1 and D2 and equal to equation (1) for D3. As a result, there is no need for a discount factor (or, equivalently, set it to 1), and the total reward is exactly equation (1).

The state variables are illustrated in Tables 1 and 2, where all the features are divided into four groups separated by the state in which those features were used:

1. Group 1: pretreatment features (before D1)
2. Group 2: post–IC-decision features (after D1 and before D2)
3. Group 3: post–CC-decision features (after D2 and before D3)
4. Group 4: primary outcomes after ND decision (after D3)

The features in Table 1 were used for the initial state s0 of the MDP and are denoted as group 1. In Table 2, the features in group 2 combined with group 1 and a 1-hot vector of D1 were used for state s1; the features in group 3 combined with groups 1-2 along with a 1-hot vector of D1 and D2 were used for state s2. As a result, the features included at each decision point represent the *complete* history of the patient up to the current treatment decision. The features in group 4 were *only* used to evaluate the reward as formulated in equation (1) and *not* used as learning features. A detailed description of all variables is given in Table S1 in Multimedia Appendix 1, and we have summarized the demographics of physicians’ decisions in Table 3.

### Preprocessing

The data set was randomly split into training (402/536, 75%) and testing (134/536, 25%) sets. To reduce the radiomic feature dimensionality (approximately 1000) [21], we applied principal component analysis and kept the 6 top components, which explain 90% of the overall feature variance. No blind assessment of the decisions or outcomes was made. Unknown human papillomavirus status was handled using a distinguished value (0). Missing values for all other covariates were handled using single imputation: median for numerical variables and mode for categorical variables. The ordinal covariates pathological grade, T (primary tumor) category, N (lymph nodes) category, AJCC staging, and prescribed chemotherapy (none, single, doublet, triplet, or quadruplet) were coded as numerical features. After these preprocessing steps, all features were rescaled to the (–1 to +1) range, as is standard in neural network (NN) training.

### DQL Neural Modeling

**Figure 2:**
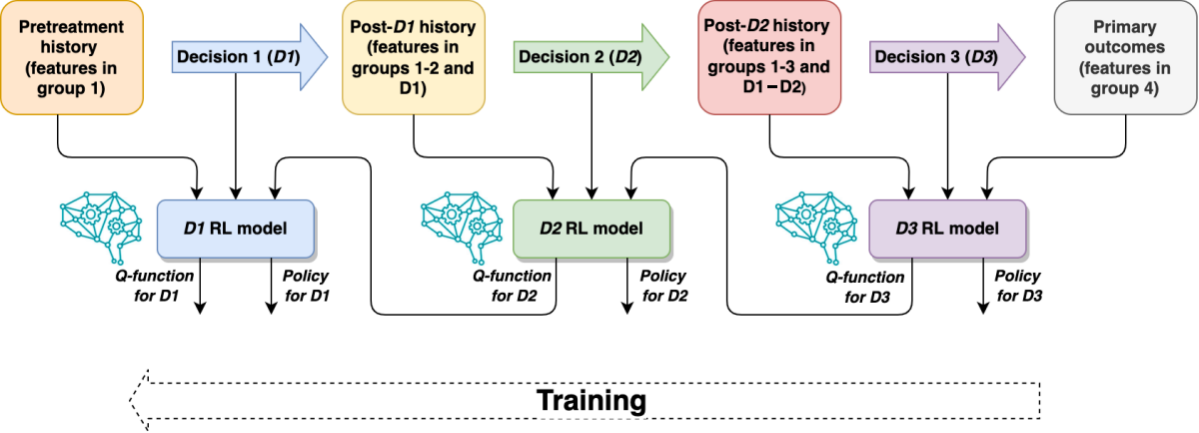
Overview of deep Q-learning model training. RL: reinforcement learning.

Figure 2 shows an overview of the training process. A separate NN model was trained for each of the decision points D1-D3. Each model was constructed recursively based on the previous model results at the subsequent decision point or the outcome (single or combined) in the case of D3. The models were trained to optimize the total rewards *without any discounting factor*, that is, the combined outcome of equation (1).

**Figure 3:**
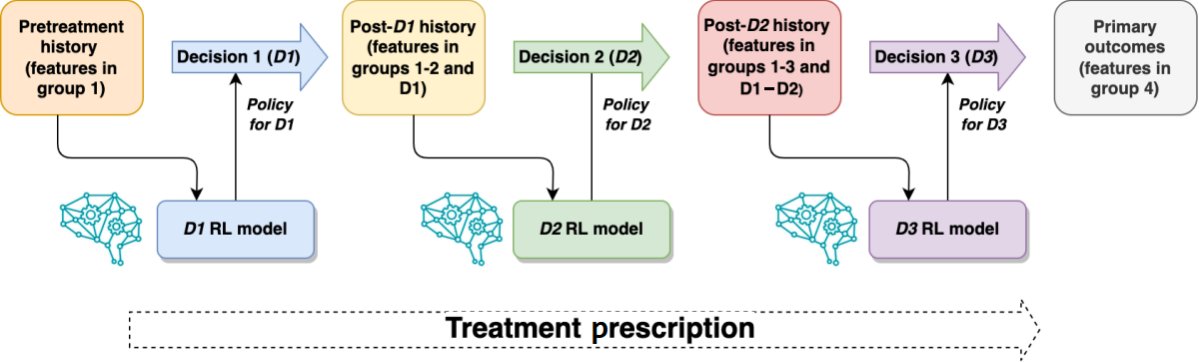
Overview of applying deep Q-learning model to make treatment prescriptions. RL: reinforcement learning.

The first model to be trained was Q3, which represents ND (D3), based on the final outcomes, the treatment decisions made in D3, and the patient’s history before D3. We tuned the learning rate so that the mean reward converged smoothly instead of fluctuating drastically. The training for D3 was terminated when the NN weights had converged. Next, the model for D2 was trained based on the result of Q3 instead of the final outcomes, and D1 was trained based on the result of Q2. The models were constructed and trained using the PyTorch framework with graphics processing unit acceleration. Once the models had been trained, they were used in a forward order, as opposed to the training order, to prescribe the optimal treatment at each decision step. This is illustrated in Figure 3.

We constructed multiple shallow-to-deep NNs with an increasing number of layers until the deepest model showed poor performance because of overfitting. We sampled 1000 separate training sets from the initial training data and trained a separate model on each of these sets, thus obtaining bootstrapped models with 95% CIs. Because of the high computational cost of bootstrapping, we will report in the *Results* section the performance of survival and toxicity under all possible numbers of hidden layers from 1 to 8, instead of performing the 5-fold cross-validation on all hyperparameters such as the number of nodes in each layer.

By prescribing an optimal treatment at each treatment junction, the DQL models constructed a *digital twin* of the decision process, with the goal of finding an optimal treatment plan that may differ from the physician’s original decisions. The trained models and the code to compute the treatment decisions have been made available on GitHub [22].

### Treatment Simulator for Evaluating DQL

**Figure 4:**
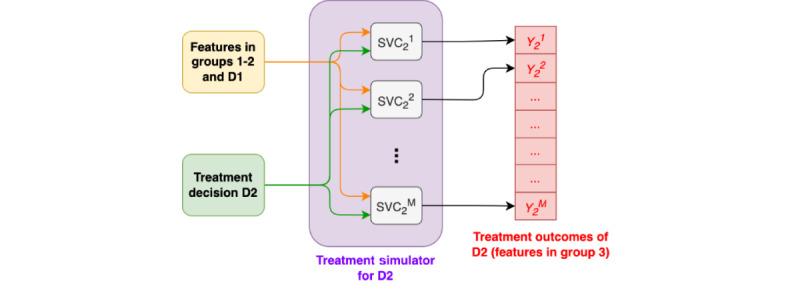
Illustration of the treatment simulator for D2. Those for D1 and D3 are similar, and their input features are from group 1 and groups 1-3, respectively. SVC: support vector classifier. D1: decision 1. D2: decision 2. D3: decision 3.

As the DQL goal is not to replicate clinicians’ decisions but to find an optimal, potentially different treatment, our evaluation includes building a treatment simulator (TS) model that, given a patient’s history and the prescribed treatment, predicts the outcome of that treatment. The TS consists of a transition model for each intermediate and final outcome measure, built using a support vector classifier (SVC). For example, in the case of D1, an SVC was trained for each group 2 feature in Table 2 to predict, as output, that feature’s value resulting from D1, and the input of these SVCs is all the Table 1 features, that is, group 1. For D2, SVC will predict for group 3 features in Table 2 using, as input, features from groups 1-2, along with a 1-hot vector of D1. The architecture is demonstrated in Figure 4. D3 was treated similarly with input features from groups 1-3, along with a 1-hot vector of D1 and D2. The full details of the SVC are provided in Table S2 in Multimedia Appendix 1. The C, *γ*, and class weights of each SVC were tuned through 5-fold cross-validation over the *F1* score on the training data because different values are needed for optimally predicting different features. Some features such as FT have quite imbalanced values, and to address this, we set the weights of each training example to be inversely proportional to the frequency of its class, hence placing more emphasis on less-common classes. The accuracy of the TS in predicting the next-stage feature value (instead of the treatment decision) was assessed with 95% CIs by using out-of-bag evaluation of 1000 models trained on stratified bootstrapped samples.

The TS serves as an in silico *digital* *twin* of the patient treatment because we can use it to dynamically simulate the patient’s in vivo course as a function-given treatment policy, without having to physically treat the patient.

### Protocol of Evaluating DQL

The DQL models were evaluated against the TS because our goal is not to replicate physicians’ decisions but to learn from the final reward and then quantitatively evaluate the treatment decisions learned by the DQL model. Such *what-if* questions are standard in off-policy evaluation in RL (in *off-policy* evaluation, one evaluates a policy without being able to implement it in the real environment). The state-of-the-art approaches fall into three categories [23]: (1) direct approach where a model of the environment is fit (same as what we do), (2) importance sampling, and (3) a combination of the 2. Importance sampling is known to suffer from high variance and thus would require a large number of samples, whereas our test set consisted of 134 patients. Similarly, Gottesman et al [24] detailed this difficulty along with several possible scenarios, but no conclusion was drawn regarding which metric to use. Yauney and Shah [25] evaluated the learned policy through simulated clinical trials, an approach identical to ours.

At the same time, to the best of our knowledge, there is no existing rule-based approach (eg, decision trees) that is suitable for this task. We note that although very generic methods such as decision trees could be customized for a single-step prediction, they do not account for the sequential nature of this decision-making process. Furthermore, ultimately, evaluating such rule-based approaches would encounter the same *what-if* questions, that is, off-policy evaluation. Indeed, this has been a long-standing open problem in RL, and we hope that our digital twin approach may provide a new *partial* solution.

Although we tested the DQL models against the TS that allows on-policy evaluation, we emphasize two important considerations in the evaluation protocol:

1. TS was not used for training. Instead, we intentionally trained DQL on a tabular observation data set of 402 patients. This is because if we did train on the TS, the learned model would overfit the simulated environment, thereby overestimating the test performance (which is also measured from the TS). This deliberate decoupling of training and testing strategy, which is also adopted by Yauney and Shah [25], is aimed specifically to ensure the fairness of the comparison. Again, note that although the TS can be directly used to optimize the policy through any model-based RL, we intentionally refrained from doing so and followed the model-free DQL. This ensures a fair testing, noting that the TS is also used in generating the testing trajectory.
2. The learned agent did not have access to the TS at test time, and the decisions were based solely on the current state. The TS was invoked only to simulate the environment, that is, generating the consequent state arising from the proposed decision and treatment, allowing the performance to be evaluated. This was consistent with the model-free nature of the DQL and ensured a fair evaluation by avoiding peeking into the real dynamics under which the test was conducted.

Incidentally, even if the TS *were* available for decision-making (that is, the planning setting), there would still be significant obstacles. In open-loop planning, a sequence of actions (3 treatment decisions in our application) is chosen *before* actually performing any of them, that is, later actions neither await nor respond to the outcome of the preceding actions. In this case, one only needs to compute 8 scores and select the optimal one. However, even in such an overly simplistic solution, one still needs to compute the expected reward, which relies on integration over the stochastic outcome of the actions, that is, states s1-s3. Mathematically, it solves




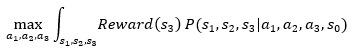




Although sampling is a natural approach to it, the high dimensionality of the state space demands a large amount of samples from the TS to accurately compute the expected reward.

In practice, closed-loop planning is clearly preferred, where later actions are chosen to best respond to the outcome of preceding decisions and treatments, leading to the mathematical optimization formulation as




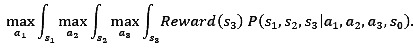




As a result, we must compute the state value (V[s]-functions) or the state-action value (Q[s,a]-functions). Because of the complexity of state space, both of them are nontrivial, even given the TS. Compared with open-loop planning, an additional layer of difficulty is incurred here because one needs to estimate 8 *functions* instead of 8 real numbers.

To summarize, this *patient treatment digital twin* approach enables us to simulate the results of applying the Q-learning models to patients and to compare the outcomes with those resulting from the clinicians’ decisions. Fairness was also upheld by not using the TS in either training or decision-making at testing time.

### Evaluation Metrics

The TS performance was evaluated by 2 accuracies without running the DQL. The *1-step* accuracy follows the trajectory from the data set and, at each of the 3 decision junctions, predicts the resulting feature value after practicing the physician’s treatment and then compares it with the ground truth outcome in the data set. In contrast, the *start-to-finish* accuracy is only concerned with the final outcome features in group 4 of Table 2. It uses the TS to generate a simulated *3-step* trajectory for each patient by following the 3 treatment decisions from the physician and compares the final outcome with the ground truth.

The DQL models were then evaluated by comparing the OS and DP rates (as computed by the TS) resulting from the DQL treatment decisions with the outcomes observed under physician treatment on a separate test set. To facilitate interpretation, we computed the similarity between each of the DQL model’s decisions and the physicians’ decisions, considering each decision point independently. This evaluation does not need the TS. To further support interpretation, the policy followed by each model was analyzed by computing the increase (or decrease) in prescription rate for each treatment decision compared with the physicians’ ad hoc prescriptions to express whether the model was more (or less) likely to prescribe a certain treatment when compared with actual physicians.

We also evaluated the DQL treatment decisions by examining compliance with the National Comprehensive Cancer Network guidelines of acceptable care [26], which state that eligible patients with advanced-stage cancer (T3-4 or N1-3) must be prescribed chemotherapy, either IC (D1) or CC (D2). These guidelines or restrictions were not explicitly imposed during model training.

We first report the performance of the TS and the simulation performance of the DQL models and compare the DQL recommendations with the physician decision process, both in terms of per-decision similarity and overall similarity, that is, averaging the similarity for each decision point for each model. To report compliance, and to ensure quality and facilitate reproducibility, we provide a formal presentation of the Transparent Reporting of a Multivariable Prediction Model for Individual Prognosis or Diagnosis checklist, formalized in Table S3 in Multimedia Appendix 1.

## Results

### Accuracy of TS Models

The complete *1-step* prediction accuracy of each TS model (with 95% CIs) is presented in Table 4. The average bootstrapped prediction accuracy of the individual TS models was 87.35% (SD 11.15%), and the median accuracy was 92.07% (IQR 13.56%). At the whole trajectory level, the average *start-to-finish* prediction accuracy on the test set outcomes was 83.21% (SD 1.54%), with 83.96% (SD 0.37%) accuracy for OS, 82.46% (SD 1.87%) for DP, 88.43% (SD 1.87%) for FT and 83.58% (SD 0.75%) for AR. Please note that these accuracy values are *neither* in terms of treatment prediction *nor* comparable with physician’s treatment D1, D2, and D3. Instead, the TS predicts the patient’s feature or state (eg, complete response or nodal) resulting from a treatment decision given in the data and compares it with the ground truth outcome in the data set. Therefore, this accuracy should not be compared with the frequency of matching physician decisions (which is 70.4%, as we will show in the *Similarity to Physicians* section).

**Table 4:** One-step prediction accuracy of treatment simulation (with 95% CIs) based on out-of-bag evaluation of 1000 stratified bootstrapped samples.

Predicted outcome	Accuracy without radiomics (%; 95% CI)	Accuracy with radiomics (%; 95% CI)
Overall survival (4 years)	78.23 (73.20-82.92)	*78.95 (74.29-83.09)* ^a^
Feeding tube (6 months)	74.37 (68.81-79.40)	*74.74 (68.53-80)*
Aspiration rate after therapy	*75 (69.38-80)*	73.96 (68.04-78.76)
Prescribed chemotherapy (single, doublet, triplet, quadruplet, none, or not otherwise specified)	*83 (77.32-87.57)*	82.77 (78.06-87.13)
Chemotherapy modification (yes or no)	*82.09 (76.96-86.34)*	80.22 (75.98- 84.82)
Dose modified	92.39 (89.23-94.95)	*94.50 (92.31-96.39)*
Dose delayed	*92.39 (89.12-95.17)*	92.35 (88.56-95.52)
Dose cancelled	91.58 (87.68-94.77)	*93.37 (90.05-96.15)*
Regimen modification	*93.54 (84.36-95.88)*	91.79 (54.7-95.05)
DLT^b^ (yes or no)	81.51 (77.25-85.42)	*81.77 (77.34-85.79)*
DLT: dermatological	*92.77 (23.95-95.29)*	90.58 (87.05-93.3)
DLT: neurological	92.17 (88.66-95.1)	*92.27 (88.83-95.26)*
DLT: gastrointestinal	89.60 (85.86-92.96)	*90.36 (86.8-93.36)*
DLT: hematological	90.10 (86.17-93.23)	*91.84 (88.02-94.47)*
DLT: nephrological	*99.03 (98-100)*	98.50 (96.55-99.52)
DLT: vascular	98.45 (96.45-100)	*98.50 (96.86-100)*
DLT: infection (pneumonia)	*98.98 (94.42-100)*	98.44 (96.37-99.50)
DLT: other	*95.08 (90.82-97.57)*	92.35 (83.17-96.98)
DLT: grade	73.85 (53.84-79.9)	*77.02 (72.55-81.48)*
No imaging (0=no and 1=yes)	100 (100-100)	100 (100-100)
Complete response, primary	83.51 (78.82-87.56)	*84.02 (79.58-88.05)*
Complete response, nodal	94.79 (90.5-97.03)	*94.82 (89.64-97.4)*
Parietal response, primary	*81.47 (76.84-86.27)*	80.32 (75.89-84.85)
Parietal response, nodal	92.93 (90-95.65)	92.93 (90.05-95.52)
Stable disease, primary	95.10 (91.96-97.84)	*96.35 (92.96-98.03)*
Stable disease, nodal	96.58 (94.47-98.05)	*97.50 (96.08-98.55)*
Concurrent chemotherapy regimen	*70 (64.68-75.27)*	65.99 (59.91-71.8)
Concurrent chemotherapy modification (yes or no)	70.53 (64.92-76.06)	*71.43 (65.68-76.68)*
Complete response, primary 2	*79.22 (23.03-85.22)*	77.35 (29.95-84.57)
Complete response, nodal 2	55.50 (49.01-61.54)	*56.25 (50-61.94)*
Parietal response, primary 2	78.92 (74.26-83.25)	*83.66 (79.90-86.60)*
Parietal response, nodal 2	52.50 (46.19-58.03)	*52.85 (46.46-58.62)*
Stable disease, primary 2	99.48 (98.46-100)	99.48 (98.41-100)
Stable disease, nodal 2	96.50 (94.12-98.04)	*96.92 (94.36-98.45)*
DLT: dermatological 2	91.99 (87.63-95.17)	*94.95 (91.53-97.07)*
DLT: neurological 2	*95.79 (5.96-97.46)*	91.97 (88.29-94.69)
DLT: gastrointestinal 2	89.74 (85.22-93.65)	*91.13 (87.50-94.06)*
DLT: hematological 2	92.71 (89.42-95.16)	*93.23 (90.10-95.57)*
DLT: nephrological 2	92.25 (88.17-97.94)	*96.53 (93.62-98.48)*
DLT: vascular 2	100 (99.45-100)	100 (99.02-100)
DLT: other 2	*93.97 (89.73-96.86)*	93.24 (89.23-96.14)

^a^Values in italics indicate whether higher accuracy is achieved by including or excluding radiomics.

^b^DLT: dose-limiting toxicity.

### Performance of DQL in OS and DP

Recall from group 4 in Table 2 that the baseline outcomes observed under physician care are 85.57% (training set) and 84.33% (test set) OS rate of staying alive and 69.65% (training set) and 76.12% (test set; absence of) DP rate.

The complete performance of all DQL models on simulated patient outcomes is presented in Table S4 in Multimedia Appendix 1. For models trained to predict both OS and DP, the selected models were the ones with 2-3 hidden layers, which outperformed physician outcomes for OS. For predicting OS on the test data, the best model with radiomics had the highest average predicted OS rate but had higher variance and a worse lower bound of 95% CI (+5.22%, 95% CI –2.26% to 10.45%) compared with the best model without radiomics (+4.48%, 95% CI –1.49% to 9.7%). For DP, models without radiomics outperformed models with radiomics in terms of both average and lower bounds in terms of simulated patient outcomes.

For the purposes of this paper, we consider the *best* model to be the 2-layer NNs without radiomic features because it had the best lower bounds on predicted OS and DP for all models, while still affording a good average performance. This model yielded a median OS improvement, compared with physicians’ results, of +2.74% (95% CI –0.25% to 6.47%; training set) and +3.73% (95% CI –0.75% to 8.96%; test set), with an absolute highest OS rate of 88.31% (95% CI 85.32%-92.04%; training set) and 88.06% (95% CI 83.58%-93.53%; test set). With respect to DP, the same 2-layer model showed a +3.98% (95% CI –1.24% to 9.2%) improvement on training data, with 73.63% (95% CI 68.41%-78.86%) of simulated patients not exhibiting DP under the model’s treatment decisions. This 2-layer model yielded a +0.75% (95% CI –4.48% to 6.72%) improvement on the test set, from the baseline 76.12% to 76.87% (95% CI 71.64%-82.84%).

**Figure 5:**
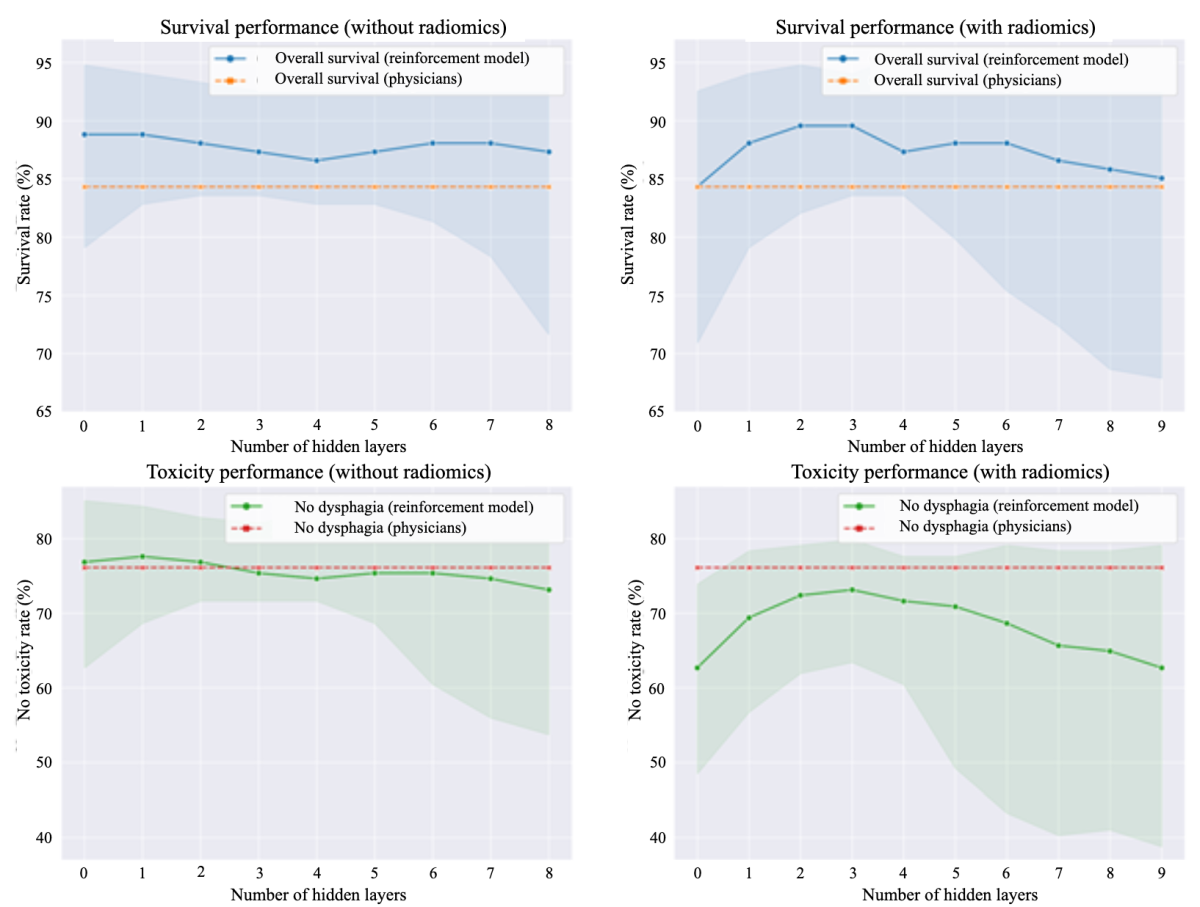
Model performance for the combined outcome (overall survival+dysphagia) models without (left) and with radiomics (right). The figure shows the performance for overall survival (top) and toxicity (dysphagia; bottom), with varying numbers of layers showing treatment simulation results on the test data.

To assess model parsimony (ie, the minimum number of layers for maintaining equivalent predictive performance), Figure 5 shows a comparison of DQL models with different numbers of layers on simulated test data for the combined outcome models (OS and DP) and with and without radiomics features. Broadly, neither simpler models with <2 layers nor models with >4 layers performed as effectively as the 2-layer model. In Figure 5, continuous lines show the average performance of the bootstrapped models, whereas highlighted areas represent the 95% CIs. Dashed lines show the empirical patient outcomes observed under the physicians’ decisions. Models with 1-4 layers had the highest performance and lower variance, whereas models with >4 layers overfit the data. Furthermore, models without radiomics had better overall performance for toxicity outcomes. Models with radiomics had slightly higher performance for OS outcomes but had higher variance and worse lower bounds than models trained without radiomics.

### Similarity to Physicians

The similar rates (with 95% CIs) with respect to physicians’ treatment *twinning*, both per-decision and overall similarity, across all models are presented in Table S5 in Multimedia Appendix 1. We reiterate that the goal of DQL is not to replicate clinicians’ decisions but to find an optimal, albeit potentially different, treatment. However, it is clearly of interest to measure the similarity as a reference. In terms of overall similarity of the in silico Q-learning treatment policies compared with those actually delivered ad hoc in vivo by physicians, our best model (OS+DP, 2 layers, and no radiomics) showed an overall 70.4% (95% CI 65.34%-73.63%) similarity to the physician decisions on the training set (ie, the model chooses the same treatment as the physicians for 70.4% of the considered treatment decisions) and 69.65% (95% CI 63.43%-73.38%) on the test set, although another model (the 3-layer OS+DP model without radiomics, which performed consistently worse in simulation for all outcomes) did show higher similarity rates.

### Compliance With National Comprehensive Cancer Network Guidelines

The distributions (with 95% CIs) of the T and N stages of patients in the test set, separated by chemotherapeutic treatment prescribed by the best-performing model, are presented in Table 5.

**Figure 6:**
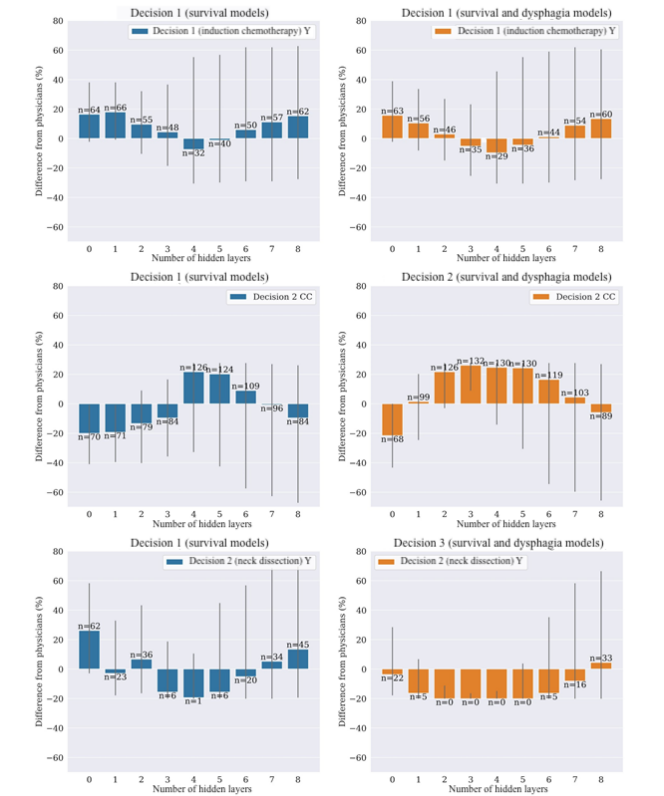
Absolute increase (or decrease) of treatment decision rate compared with physicians’ decisions. The plots refer to decisions 1 (top), 2 (middle), and 3 (bottom) on the test set and for models considering only overall survival as an outcome measure (left) or overall survival+dysphagia (right) without radiomics. Y: yes.

The rates at which models choose a certain policy compared with the physicians’ treatment rate at each decision point are shown in Figure 6. Gray lines represent the 95% CIs. The numbers on top of the bars show for how many patients (out of 134) the Q-learning model recommended that treatment. The IC prescription rate varies in a similar way between OS and OS+DP, CC is significantly more frequent in OS+DP models, whereas ND is consistently less frequent in OS+DP. The best-performing model in terms of simulated outcomes (OS+DP, 2 layers, and no radiomics) had a higher IC (D1) rate (2.99% increase in prescription rate compared with physicians’ prescriptions for 46 patients, 95% CI –14.93% to 26.88%) and one of the highest CC (D2) rates (21.64% increase, 126 patients, 95% CI –2.99% to 27.61%), as well as the lowest ND (D3) rate (20.15% decrease, 0 patients, 95% CI –20.15% to –11.19%).

**Table 5:** Tumor stage demographics of patients based on the chemotherapeutic treatment decisions of the best-performing model (n=134).

Demographics			Chemotherapy					No chemotherapy, no induction chemotherapy, radiotherapy alone (%; 95% CI)
			Induction chemotherapy			No induction chemotherapy, concurrent chemotherapy (%; 95% CI)		
			Concurrent chemotherapy (%; 95% CI)	Radiotherapy alone (%; 95% CI)				
**T^a^ category**								
		T1	23.08 (0-65.38)	3.85 (0-26.92)	69.23 (26.92- 96.15)		0 (0-23.08)	
		T2	25.40 (6.35-55.56)	3.17 (0-22.22)	66.67 (38.06-88.89)		0 (0-20.63)	
		T3	32 (8-64.1)	4 (0-28)	60 (28-88)		0 (0-16)	
		T4	36.84 (5.26-84.21)	5.26 (0-31.58)	52.63 (10.53-94.74)		0 (0-15.79)	
		Tx^b^	0 (0-100)	0 (0-100)	100 (0-100)		0 (0-100)	
**N^c^ category**								
	N0^d^		20 (0-100)	0 (0-40)	60 (0-100)		0 (0-40)	
	N1		17.39 (0-73.91)	0 (0-30.43)	73.91 (17.39-95.65)		0 (0-21.74)	
	N2		29.41 (9.80-56.89)	3.92 (0-22.55)	62.75 (36.27-81.37)		0 (0-17.65)	
	N3		25 (0-100)	0 (0-50)	50 (0-100)		0 (0-25)	
**N category (8th edition^e^)**								
	N0		16.67 (0-100)	0 (0-33.33)	66.67 (0-100)		0 (0-33.33)	
	N1		22.06 (2.94-60.29)	2.94 (0-26.47)	70.59 (30.88-92.65)		0 (0-22.06)	
	N2		33.93 (8.93-69.64)	3.57 (0-26.79)	58.93 (25-83.93)		0 (0-14.29)	
	N3		25 (0-100)	0 (0-50)	50 (0-100)		0 (0-25)	

^a^T: primary tumor.

^b^Tx: no information about the primary tumor or it cannot be measured.

^c^N: lymph nodes.

^d^N0: nearby lymph nodes do not contain cancer.

^e^American Joint Committee on Cancer’s Cancer Staging Manual, 8th edition.

### Computational Cost

The training time for a single DQL model did not significantly vary between shallower and deeper NNs and was just a few minutes on average for a complete model. With 1000-sample bootstrapping, the training time was accordingly longer, costing >24 hours to generate the results shown in Figure 5. However, these models are computationally inexpensive and can be deployed virtually in real time. Because of the computational cost of bootstrapping, we only used 5-fold cross-validation to tune the TS hyperparameters using SVCs, whereas for the other backbone hyperparameters such as the number of layers, we opted to report the performance of OS and DP under all possible numbers of hidden layers from 1 to 8 instead of performing cross-validation.

### Clinical Case Review

As there is no practical way of verifying counterfactual *what-if* scenarios in actual patient care, we performed a post hoc case review of selected divergence of the policy prediction from delivered care. We herein report 2 case representative studies of patient-specific DQL treatment decisions—one differed from, whereas the other mostly concurred with, the original decisions of the treatment team of physicians—with discussion and input from oncologists at the MDACC. These 2 case studies were selected by performing 1000 bootstrapped trials on the entire data set (randomly partitioning it into training and testing), then recording the frequency with which the DQL prediction agreed with the physician’s (among the 1000 bootstrapped trials). We used the smallest geometric mean value to select the first case study. The second case study was selected for high values of agreement rates in D2 and D3 but a low value of agreement rates in D1.

The patient in the first case study differed in every decision: the treatment sequence prescribed by their clinician team was D1: IC, D2: RT, and D3: ND, whereas the DQL sequence was D1: not IC, D2: CC, and D3: not ND. During our discussion, upon retrieving and examining the medical records, the oncologists described this case as having “a very unique and strange presentation” with bilateral disease involving the retropharyngeal lymph node (RPN). As the MDACC has historically associated RPN involvement with increased metastatic risk in published series [27,28], the patient was prescribed IC in D1 as part of an informal local policy for cases with perceived high risk of distant metastases or unsalvageable nodal failure (eg, retropharyngeal recurrence). The patient exhibited a substantive response with respect to the response evaluation criteria in solid tumors, wherein the primary tumor volume and index RPN had clinical complete response; therefore, in D2, the physicians prescribed RT alone. The patient exhibited a sizable response again, this time to the primary tumor. After RT, there was a notation by the radiologist of a negative positron emission tomography scan (eg, no evidence of metabolic uptake); however, the nodal remnant was evident and “malignancy could not be excluded.” Consequently, in D3, the physicians prescribed, as a precaution, a completion ND on the lymph node but found no cancer, only necrotic tissue. In the oncologists’ assessment, the DQL sequence would approximate typical standard of care more than the delivered treatment in terms of general community practice. In this case, the MDACC team altered the treatment based on additional *local* information related to the RPN. However, in their assessment, most other centers would not alter treatment based on this *typically not collected* information because RPN status is not a formal component of staging materials or risk categories for oropharyngeal cancer [29,30] nor of AJCC staging systems [31], and many, if not most, practices would treat this case as concurrent chemoradiotherapy. In summary, this was an extremely unusual, unique case where additional unannotated *local* information made the difference between the DQL and the prescriber’s sequence. Future work that includes nodal involvement methodology [3,4] not currently reflected in the AJCC 8th Edition could address this type of borderline case.

The patient we considered in the second case study featured disagreement only in the first decision. The treatment sequence prescribed by their clinician team was D1: not IC, D2: CC, and D3: not ND, whereas the DQL sequence was D1: IC, D2: CC, and D3: not ND. Upon examining the medical records, the oncologists noted that the patient had only 1 functioning kidney; therefore, in the first stage, the team decided to prescribe a low-dose chemotherapy regimen treatment as a precaution to prevent renal injury [32]. In their assessment, our dyad system performed well, given the input specifications of this case, and the difference in therapy selection was attributed to occult (but clinically meaningful) comorbid disease variables not included in the decision platform that influenced the physicians’ process.

Overall, the physician review in both these instances that we investigated in detail suggests that, in the absence of specific *local* practices or occult clinical features not included in this decision platform, the DQL recommendation would have been a good strategy and that the dyad provided “clinically acceptable recommendations.”

## Discussion

### Principal Findings

The high average, median, and overall accuracies provided by the TS in predicting the outcomes of treatments indicate that the TS is a valid digital twin for the treatment process when predicting the outcome of a treatment sequence. Our results also indicate that the Q-learning models indeed capture the nature of the dynamic treatment problem and provide a valid solution. Our models showed consistent improvements for all the outcome features taken into account, as well as moderate similarity to physicians’ decisions. Overall, these results indicate that DQL modeling can serve as a digital twin of the treatment decision process and TS modeling can serve as a digital twin of the patient treatment. When combined, DQL and TS constitute a valid patient-physician *digital twin dyad* for optimal policy determination in sequential systemic and locoregional therapy of oropharyngeal squamous carcinomas.

Furthermore, our results show that the DQL models that consider OS+DP outperform models considering only OS in terms of simulated survival rate. As the absence of DP (FT or AR) symptoms is positively correlated with OS, maximizing these indirectly helps maximize OS-model performance as well.

Moreover, OS+DP models show higher similarity to actual physician decisions because they represent a finer-grained approximation of the decision process than models that include only OS as an outcome, including more of the features considered by the physician when choosing an optimal treatment.

Surprisingly, given the abundance of data on radiomics models for head and neck cancer [13,33-40], Q-learning models *without* radiomics yielded a better performance than models that included radiomics, in terms of simulated outcomes and variance, for both training and testing data. The most evident example is given by the simulated DP: none of the models trained with radiomics features managed to improve the outcome observed after physicians’ treatment in the test set (Figure 5, bottom right). From these results, the addition of textural features *failed* to improve model performance and instead significantly increased the performance variance of the bootstrapped models.

Our findings also justify the choice of a deep NN model instead of a regular linear model: whereas by using DQL we reduce model parsimony, we can see that the results of the linear models (ie, the 0–hidden-layers NNs) are comparatively suboptimal to deeper models in terms of simulated performance, CI variance, and similarity.

Furthermore, per Table 5, the best-performing model did not violate the standard of care regarding chemotherapeutic treatment of patients because all patients with stages T3-4 or N1-3 cancer were prescribed either IC (D1) or CC (D2), with most of them being prescribed at least CC, showing that clinical applicability was maintained.

When comparing OS-only models with OS+DP models, the prescription rates presented in Figure 5 showed 2 separate trends for the 2 categories: whereas the IC (D1) prescription rate varied similarly for the OS and OS+DP models, the rates of CC (D2) and ND (D3) were significantly different between the 2 categories. In general, OS+DP models tended to have a higher rate of CC (D2) and a lower rate of ND (D3). In other words, *models that also consider toxicity as an outcome measure balanced a more aggressive chemotherapeutic treatment with a lower rate of ND*, which is consistent with the known positive correlation between surgery and DP symptoms such as FT and AR.

### Limitations

Although the proposed approach was shown to be effective in dynamically selecting optimal treatment strategy for patients with oropharyngeal squamous cell carcinoma, it is not without limitations. Because of the retrospective nature of the data set, our Q-learning models had to be evaluated through the TS, a supervised learning model, which might be seen as self-referential. However, the TS is a necessary approach before prospective application because evaluating the models based on physician similarity alone would not reflect the purpose of DQL. Intuitively, the goal of DQL is not to replicate the decisions taken by physicians in the data set but to learn from these decisions and their effect to discern between optimal and nonoptimal choices with respect to a given outcome measure.

Furthermore, because we train our *digital twin dyad* on a representative cohort from a single cancer center (MDACC), the physician decision or prescribing heuristics reflected may not be fully generalizable to other facilities with other practitioners. However, the physician prescriptions at the MDACC are aligned with the state of the art in the field. In particular, we note that whereas 2 studies [41,42] have questioned the relevance of IC to treatment, both studies have failed to accrue and thus are null. Our modeling approach could conceivably be implemented and extended to generate similar *digital twin dyad*s across other or multiple institutions.

### Conclusions

In conclusion, we constructed a DQL modeling approach to make optimized sequential treatment decisions based on a set of desired outcomes in head and neck cancer therapy and paired it with a simulation of the treatment process for evaluation purposes. This modeling approach represents, to our knowledge, the first application of DQL with simulation as a *digital twin dyad* to simultaneously represent both s*tate-specific patient data* and *physician or prescriber policies* for head and neck squamous carcinoma. Furthermore, this work is the first reported implementation of DQL for DP and OS composite-outcome modeling. Our approach further demonstrates the technical feasibility of such a *digital twin dyad* and provides a benchmarking data set and relevant code for model dissemination, site-specific implementation, and iterative model improvement. Carrying out a prospective clinical study application could further confirm the validity of this approach as part of the standard of care.

## Appendix

Multimedia Appendix 1 Supplementary Tables 1-5.

## Abbreviations

AJCC: American Joint Committee on Cancer
AR: aspiration rate
CC: concurrent chemotherapy
DP: dysphagia
DQL: deep Q-learning
FT: feeding-tube dependence
IC: induction chemotherapy
MDACC: MD Anderson Cancer Center
MDP: Markov decision process
ND: neck dissection
NN: neural network
OS: overall survival
RL: reinforcement learning
RPN: retropharyngeal lymph node
RT: radiotherapy
SVC: support vector classifier
TS: treatment simulator

## Data Availability

The data set used in the data analysis is publicly available [43].
