# Supplementary material for: Optimal Treatment Selection in Sequential Systemic and Locoregional Therapy of Oropharyngeal Squamous Carcinomas: Deep Q-Learning With a Patient-Physician Digital Twin Dyad
Source: J Med Internet Res. 2022 Apr 20;24(4):e29455. doi: 10.2196/29455 (PMC9069283; doi:10.2196/29455)
Supplement: Multimedia Appendix 1 [file jmir_v24i4e29455_app1.docx]

**Supplement: Table of contents**

**Table S1: Descriptions of the variables considered in the treatment simulator. Variables that were accessed after each decision were reassessed after each treatment decision and treated as separate variables. 2**

**Tabel S2: Training details for treatment simulator 4**

**Table S3: TRIPOD checklist 6**

**Table S4: Absolute improvement over physicians’ results with treatment simulation, per model, without and with radiomics, and for different model outcomes (OS vs. OS+DP). 12**

**Table S5: Similarity between the model prediction and the original treatment decision on training and testing data, without radiomics and with radiomics, and for different model outcomes (OS and OS+DP). 16**

#

# Table S1: Descriptions of the variables considered in the treatment simulator. Variables that were accessed after each decision were reassessed after each treatment decision and treated as separate variables.

| Feature | Description | Data Type |
| --- | --- | --- |
| **Group 4: Primary Outcomes (after D3)** | | |
| Overall Survival (OS) | If the patient survived until 4 months after treatment | Boolean (1 = survived) |
| Aspiration Rate (AR) | If the patient required aspiration of fluid in the lungs at 6 months after treatment ceased. | Boolean (1 = survived) |
| Feeding Tube (FT) | If the patient required a feeding tube at 6 months after treatment ceased. | Boolean (1 = survived) |
| Dysphagia (DP) | If the patient required a feeding tube or aspiration of fluid in the lungs at 6 months after treatment. | Boolean (1 = survived) |
| **Group 3: Post-Concurrent Chemotherapy variables Only (after D2 and before D3)** | | |
| CC Regimen | Type of chemotherapy agent given during radiotherapy delivery | Categorical   - None - Platinum Based - Cetuximab Based - Other |
| CC modification | Whether a modification was done to concurrent chemotherapy | Boolean (1 = Yes) |
| DLT Grade | The grade of toxicity that causes regimen modification based on their severity, based on Common Terminology Criteria for Adverse Events (CTCAE version 4.0). | Ordinal   - 0 (lowest) - 1 - 2 - 3 - 4 (highest) |
| DLT Dermatological | Presence of skin toxicity | Boolean (1 = Yes) |
| DLT Neurological | Presence of nerve toxicity | Boolean (1 = Yes) |
| DLT Gastrointestinal | Presence of stomach or intestinal toxicity | Boolean (1 = Yes) |
| DLT Hematological | Presence or absence of blood toxicity | Boolean (1 = Yes) |
| DLT Nephrological | Presence or absence of renal (kidney) toxicity | Boolean (1 = Yes) |
| DLT Vascular | Presence of vascular toxicity | Boolean (1 = Yes) |
| DLT Infection | Presence of infection-related toxicity (pneumonia) | Boolean (1 = Yes) |
| DLT Other | Presence of dose-limiting toxicity not otherwise specified | Boolean (1 = Yes) |
| Complete Response Primary (CR Primary) | Complete response in primary tumor following each decision, accessed radiologically. | Boolean (1 = Yes) |
| Complete Response Nodal (CR Nodal) | Complete response in regional nodal sites following each decision, accessed radiologically. | Boolean (1 = Yes) |
| Parietal Response Primary (PR Primary) | Parietal response in primary tumor following each decision, accessed radiologically. | Boolean (1 = Yes) |
| Parietal Response Nodal (PR Nodal) | Parietal response in regional nodal sites following each decision, accessed radiologically. | Boolean (1 = Yes) |
| Stable Disease Primary (SD Primary) | Stable disease in primary tumor following each decision, accessed radiologically. | Boolean (1 = Yes) |
| Stable Disease Nodal (SD Primary) | Stable disease in regional nodal sites following each decision, accessed radiologically. | Boolean (1 = Yes) |
| **Group 2: Post-Induction Therapy variables Only (after D1 and before D2)** | | |
| Prescribed Chemo | Type of induction chemotherapy agents given as a combination regimen to a patient. | Categorical   - Doublet: two drugs - Triplet: three drugs - Quadruplet: four drugs - None: no chemotherapy agents - NOS (not otherwise specified): patient received chemotherapy, but no record of agents given |
| Chemo Modification | Whether a modification was done to concurrent chemotherapy | Boolean (1 = Yes) |
| Modification Type | Type of modification done to the chemotherapy regimen | Categorical   - No Dose Adjustment - Dose Modified - Dose Delayed - Dose Cancelled - Dose Delayed & Modified - Regimen Modification - Unknown |
| Imaging | If a radiological assessment was done for a patient following decision 1 (induction chemo Y/N) | Boolean (1 = Yes) |
| **Treatment Decisions** | | |
| Induction Chemo | If a patient receives induction chemotherapy as part of their treatment. | Boolean (1 = Yes) |
| CC/RT Alone | Whether the patient receives a concurrent chemo-radiotherapy in addition to chemo alone | Boolean (1 = Concurrent chemo-radiotherapy) |
| Neck Dissection | Whether the patient undergoes a neck dissection | Boolean (1 = Yes) |

# Table S2: Training Details for Treatment Simulator

Detailed training parameters of each SVC model for primary and secondary outcomes used in the treatment simulator: kernel type, C value, degree (only valid for polynomial kernel), use of radiomics features, and prediction accuracy with 95% confidence intervals. All models used an automatically determined gamma and balanced class weights, i.e., the weight of each training example was inversely proportional to its class’ frequency, hence placing more emphasis on less common classes. Primary outcomes are in bold. Variables with a 2 denote that these were reassessed after decision 2.

| *Outcome* | *C* | *Kernel* | *Degree* | *Radiomics (Y/N)* | *Accuracy (95% CI)* |
| --- | --- | --- | --- | --- | --- |
| **Primary Outcomes** | | | | | |
| **Overall Survival (4 Years)** | 100 | gaussian | - | Y | 78.95% (74.29%, 83.09%) |
| **Feeding tube (6 months)** | 10 | gaussian | - | Y | 74.74% (68.53%, 80.0%) |
| **Aspiration rate (6 months)** | 10 | gaussian | - | N | 75.0% (69.38%, 80.0%) |
| **Post-Induction Therapy variables (after D1 and before D2)** | | | | | |
| Prescribed Chemo (Single/doublet/triplet/quadruplet/none/NOS) | 3 | polynomial | 5 | N | 83.0% (77.32%, 87.57%) |
| Chemo Modification (Y/N) | 1 | gaussian | - | N | 82.09% (76.96%, 86.34%) |
| Dose modified | 100 | polynomial | 20 | Y | 94.5% (92.31%, 96.39%) |
| Dose delayed | 60 | gaussian | - | N | 92.39% (89.12%, 95.17%) |
| Dose cancelled | 100 | gaussian | - | Y | 93.37% (90.05%, 96.15%) |
| Regimen modification | 3 | polynomial | 20 | N | 93.54% (84.36%, 95.88%) |
| DLT (Y/N) | 10 | polynomial | 10 | Y | 81.77% (77.34%, 85.79%) |
| DLT_Dermatological | 2 | polynomial | 15 | N | 92.77% (23.95%, 95.29%) |
| DLT_Neurological | 100 | gaussian | - | Y | 92.27% (88.83%, 95.26%) |
| DLT_Gastrointestinal | 200 | polynomial | 10 | Y | 90.36% (86.8%, 93.36%) |
| DLT_Hematological | 10000 | polynomial | 10 | Y | 91.84% (88.02%, 94.47%) |
| DLT_Nephrological | 2 | polynomial | 20 | N | 99.03% (98.0%, 100.0%) |
| DLT_Vascular | 4 | gaussian | - | Y | 98.5% (96.86%, 100.0%) |
| DLT_Infection (Pneumonia) | 1 | polynomial | 15 | N | 98.98% (94.42%, 100.0%) |
| DLT_Other | 3 | gaussian | - | N | 95.08% (90.82%, 97.57%) |
| DLT_Grade | 1000 | polynomial | 15 | Y | 77.02% (72.55%, 81.48%) |
| No imaging (0=N, 1=Y) | 1 | gaussian | - | Y | 100.0% (100.0%, 100.0%) |
| SD Primary | 4 | polynomial | 10 | Y | 84.02% (79.58%, 88.05%) |
| SD Nodal | 1000 | polynomial | 5 | Y | 94.82% (89.64%, 97.4%) |
| PR Primary | 2 | gaussian | - | N | 81.47% (76.84%, 86.27%) |
| PR Nodal | 1 | gaussian | - | N | 92.93% (90.0%, 95.65%) |
| SD Primary | 10 | gaussian | - | Y | 96.35% (92.96%, 98.03%) |
| SD Nodal | 3 | polynomial | 15 | Y | 97.5% (96.08%, 98.55%) |
| **Post-Concurrent Chemotherapy variables (after D2 and before D3)** | | | | | |
| CC Regimen | 10000 | gaussian | - | N | 70.0% (64.68%, 75.27%) |
| CC modification (Y/N) | 10000 | gaussian | - | Y | 71.43% (65.68%, 76.68%) |
| CR Primary 2 | 2 | polynomial | 30 | N | 79.22% (23.03%, 85.22%) |
| CR Nodal 2 | 3 | sigmoid | - | Y | 56.25% (50.0%, 61.94%) |
| PR Primary 2 | 100000 | polynomial | 60 | Y | 83.66% (79.9%, 86.6%) |
| PR Nodal 2 | 10000 | polynomial | 30 | N | 52.85% (46.46%, 58.62%) |
| SD Primary 2 | 1 | gaussian | - | Y | 99.48% (98.46%, 100.0%) |
| SD Nodal 2 | 100 | gaussian | - | Y | 96.92% (94.36%, 98.45%) |
| DLT_Dermatological 2 | 1000 | polynomial | 30 | Y | 94.95% (91.53%, 97.07%) |
| DLT_Neurological 2 | 10 | polynomial | 50 | N | 95.79% (5.96%, 97.46%) |
| DLT_Gastrointestinal 2 | 10000 | polynomial | 10 | Y | 91.13% (87.5%, 94.06%) |
| DLT_Hematological 2 | 1000 | polynomial | 30 | Y | 93.23% (90.1%, 95.57%) |
| DLT_Nephrological 2 | 10 | gaussian | - | Y | 96.53% (93.62%, 98.48%) |
| DLT_Vascular 2 | 1 | gaussian | - | N | 100.0% (99.45%, 100.0%) |
| DLT_Other 2 | 2 | polynomial | 30 | N | 93.97% (89.73%, 96.86%) |

#

# Table S3: TRIPOD checklist

This checklist is adapted from the Tripod checklist for predictive ML models, taking into account that our model is not a predictive one, but one that seeks to optimize a treatment sequence.

| **Section/Topic** | **Item** | **Checklist item** | **Page** |
| --- | --- | --- | --- |
| **Title and abstract** | | | |
| Title | 1 | Identify the study as developing the model, the target population, and the model purpose | 1 |
| Abstract | 2 | Provide a summary of objectives, study design, setting, participants, sample size, variables, outcome, statistical analysis, results, and conclusions. | 1-3 |
| **Introduction** | | | |
| Background and objectives | 3a | Explain the medical context and rationale for developing or validating the model, including references to existing models. | 4-6 |
|  | 3b | Specify the objectives, including whether the study describes the development or validation of the model or both. | 6 |
| **Methods** | | | |
| Source of data | 4a | Describe the study design or source of data (e.g., randomized trial, cohort, or registry data), separately for the development and validation data sets, if applicable | 7-8 |
|  | 4b | Specify the key study dates, including start of accrual; end of accrual; and, if applicable, end of follow-up. | 7 |
| Participants | 5a | Specify key elements of the study setting (e.g., primary care, secondary care, general population) including number and location of centres. | 7-8 |
|  | 5b | Describe eligibility criteria for participants. | 7-8 |
|  | 5c | Give details of treatments received, if relevant. | 7, 9-13 (Table 1-3) |
| Outcome | 6a | Clearly define the decisions taken and the outcome maximized by the model, including how and when assessed. | 5 (Figure 1), 7-8, 11-13 (Table 2-3) |
|  | 6b | Report any actions to blind assessment of the outcome to be maximized or decisions to be taken. | 13 |
| Predictors | 7a | Clearly define all predictors used in developing or validating the multivariable prediction model, including how and when they were measured. | Manuscript: 9-13 (Tables 1-3)  Supplement: e21-e23 (eTable 5) |
|  | 7b | Report any actions to blind assessment of features. | 13 |
| Sample size | 8 | Explain how the study size was arrived at | 7 |
| Missing data | 9 | Describe how missing data were handled (e.g., complete-case analysis, single imputation, multiple imputation) with details of any imputation method. | 13-14 |
| Statistical analysis method | 10a | Describe how variables were handled in the analyses | 13-14 |
|  | 10b | Specify type of model, all model-building procedures (including any feature selection), and method for internal validation. | Manuscript: 14-19, 14 (Figure 2), 15 (Figure 3), 17 (figure 4)  Supplement: e12-e13 (eTable 3) |
|  | 10d | Specify all measures used to assess model performance and, if relevant, to compare multiple models. | 17-21 |
| Risk groups | 11 | Provide details on how risk groups were created, if done. | Not applicable |
| **Results** | | | |
| Participants | 13a | Describe the flow of participants through the study, including the number of participants with and without the outcome and, if applicable, a summary of the follow-up time. A diagram may be helpful. | 13 (Table 4), 14 (Figure 2), 15 (Figure 3), 27 (Table 5) |
|  | 13b | Describe the characteristics of the participants (basic demographics, clinical features, available predictors), including the number of participants with missing data for predictors and outcome. | Manuscript: 9, 9-13 (Tables 1-3), 14  Supplement: e20-e22 (eTable 5) |
| Model development | 14a | Specify the number of participants and outcome events in each analysis. | Manuscript: 7, 13 (Figure 3), 22-24 (Table 4), 24-25, 27, 29, 30 (Figure 6)  Supplement: e6-e11 (eTable 2) |
|  | 14b | If done, report the unadjusted association between each candidate predictor and outcome. | Not applicable |
| Model specification | 15a | Present the full model to allow usage for individuals (i.e., all regression coefficients, and model intercept or baseline survival at a given time point) | Manuscript: 15 and [19]  Supplement: e12-13 (eTable 3) |
|  | 15b | Explain how to use the model | 14-16 |
| Model performance | 16 | Report performance measures (with CIs) for the model. | Manuscript: 21, 22-24 (Table 4), 24-26, 26 (Figure 5), 27-29, 27-28 (Table 5)  Supplement: e2-e5 (eTable 1), e6-e11 (eTable 2), e12-e13 (eTable 3) |
| **Discussion** | | | |
| Limitations | 18 | Discuss any limitations of the study (such as non-representative sample, few events per variable, missing data). | 32, 35 |
| Interpretation | 19b | Give an overall interpretation of the results, considering objectives, limitations, and results from similar studies, and other relevant evidence. | 31-35 |
| Implications | 20 | Discuss the potential clinical use of the model and implications for future research | 33, 37 |
| **Other information** | | | |
| Supplementary information | 21 | Provide information about the availability of supplementary resources, such as study protocol, Web calculator, and data sets. | 44 and [32] |
| Funding | 22 | Give the source of funding and the role of the funders for the present study. | 38-39 |

# Table S4: Absolute improvement over physicians’ results with treatment simulation, per model, without and with radiomics, and for different model outcomes (OS vs. OS+DP).

# In brackets are the 95% confidence intervals. Best results are shown in bold.

| *Model Outcome* | *Radiomics* | *Hidden Layers* | *Training* | | *Testing* | |
| --- | --- | --- | --- | --- | --- | --- |
|  |  |  | *Overall Survival (4 Years)* | *Dysphagia* | *Overall Survival (4 Years)* | *Dysphagia* |
| Overall Survival | No | 0 | -0.25% (-14.43%, +6.97%) |  | +0.75% (-12.69%, +8.96%) |  |
|  |  | 1 | +3.73% (-6.23%, +8.21%) |  | +4.48% (-5.22%, +10.45%) |  |
|  |  | 2 | +2.24% (-9.45%, +7.96%) |  | +2.99% (-9.7%, +9.7%) |  |
|  |  | 3 | +3.73% (-2.99%, +8.71%) |  | +4.48% (-2.26%, +9.7%) |  |
|  |  | 4 | +1.49% (-2.24%, +8.96%) |  | +2.24% (-1.49%, +10.47%) |  |
|  |  | 5 | +1.74% (-4.23%, **+9.2%**) |  | +2.99% (-3.73%, +10.45%) |  |
|  |  | 6 | +1.49% (-7.71%, +8.46%) |  | +2.99% (-6.72%, **+11.19%**) |  |
|  |  | 7 | +1.49% (-12.44%, +8.46%) |  | +2.99% (-9.72%, +10.45%) |  |
|  |  | 8 | +1.0% (-17.67%, +8.46%) |  | +2.24% (-14.93%, +10.45%) |  |
|  | Yes | 0 | -0.5% (-12.46%, +6.22%) |  | -0.75% (-13.43%, +8.23%) |  |
|  |  | 1 | +2.74% (-4.98%, +7.46%) |  | +3.73% (-5.97%, +9.7%) |  |
|  |  | 2 | +2.99% (-2.25%, +7.46%) |  | +3.73% (-2.99%, +8.96%) |  |
|  |  | 3 | +1.74% (-1.99%, +6.97%) |  | +3.73% (-1.49%, +9.7%) |  |
|  |  | 4 | +1.49% (-2.49%, +7.71%) |  | +3.73% (-1.49%, +10.45%) |  |
|  |  | 5 | +1.49% (-3.73%, +8.46%) |  | +3.73% (-4.48%, **+11.19%**) |  |
|  |  | 6 | +1.74% (-6.22%, +7.96%) |  | +2.99% (-7.48%, **+11.19%**) |  |
|  |  | 7 | +1.49% (-10.71%, +7.71%) |  | +2.99% (-12.69%, +10.45%) |  |
|  |  | 8 | +0.75% (-12.94%, +7.46%) |  | +1.49% (-13.43%, +10.45%) |  |
|  |  | 9 | +0.5% (-15.93%, +7.21%) |  | +1.49% (-16.42%, +9.7%) |  |
| Overall Survival and Dysphagia | No | 0 | +3.73% (-5.47%, +8.46%) | **+5.97%** (-4.73%, +12.44%) | +4.48% (-5.22%, +10.45%) | +0.75% (-13.43%, **+8.97%**) |
|  |  | 1 | +3.73% (-1.24%, +7.96%) | +4.98% (-1.75%, +11.19%) | +4.48% (-1.49%, +9.7%) | **+1.49%** (-7.46%, +8.23%) |
|  |  | 2 | +2.74% (**-0.25%**, +6.47%) | +3.98% (**-1.24%**, +9.2%) | +3.73% (**-0.75%**, +8.96%) | +0.75% (**-4.48%**, +6.72%) |
|  |  | 3 | +1.74% (-1.24%, +6.47%) | +1.99% (-2.74%, +8.96%) | +2.99% (**-0.75%**, +8.21%) | -0.75% (**-4.48%**, +5.97%) |
|  |  | 4 | +1.0% (-1.74%, +8.21%) | +1.24% (-2.99%, +11.95%) | +2.24% (-1.49%, +9.7%) | -1.49% (**-4.48%**, +7.46%) |
|  |  | 5 | +1.49% (-1.99%, +8.96%) | +1.74% (-3.98%, +12.94%) | +2.99% (-1.49%, **+11.19%**) | -0.75% (-7.46%, +8.21%) |
|  |  | 6 | +2.24% (-2.99%, +8.96%) | +1.99% (-7.71%, +12.94%) | +3.73% (-2.99%, **+11.19%**) | -0.75% (-15.67%, +8.21%) |
|  |  | 7 | +2.24% (-7.72%, +8.96%) | +2.24% (-11.69%, **+13.43%**) | +3.73% (-5.99%, +10.47%) | -1.49% (-20.15%, **+8.97%**) |
|  |  | 8 | +1.74% (-14.43%, +8.46%) | +1.24% (-12.2%, +12.69%) | +2.99% (-12.69%, +10.45%) | -2.99% (-22.39%, +8.96%) |
|  | Yes | 0 | -0.5% (-12.2%, +5.72%) | -6.97% (-17.91%, +1.99%) | 0.0% (-13.43%, +8.21%) | -13.43% (-27.63%, -2.24%) |
|  |  | 1 | +2.74% (-5.47%, +7.21%) | -2.74% (-11.95%, +5.22%) | +3.73% (-5.22%, +9.7%) | -6.72% (-19.4%, +2.24%) |
|  |  | 2 | **+4.23%** (-2.0%, +7.96%) | 0.0% (-8.46%, +6.22%) | **+5.22%** (-2.26%, +10.45%) | -3.73% (-14.18%, +2.99%) |
|  |  | 3 | +3.73% (-1.24%, +7.47%) | -0.25% (-7.96%, +6.47%) | **+5.22%** (**-0.75%**, +9.7%) | -2.99% (-12  .71%, +3.73%) |
|  |  | 4 | +1.0% (-2.24%, +8.21%) | -4.23% (-11.19%, +4.73%) | +2.99% (**-0.75%**, **+11.19%**) | -4.48% (-15.67%, +1.49%) |
|  |  | 5 | +1.74% (-3.73%, +8.21%) | -4.48% (-20.15%, +5.73%) | +3.73% (-4.5%, +10.45%) | -5.22% (-26.88%, +1.49%) |
|  |  | 6 | +1.74% (-6.98%, +7.96%) | -4.98% (-22.39%, +6.72%) | +3.73% (-8.96%, **+11.19%**) | -7.46% (-32.84%, +2.99%) |
|  |  | 7 | +1.24% (-11.2%, +7.46%) | -6.09% (-24.38%, +6.47%) | +2.24% (-11.96%, +10.45%) | -10.45% (-35.82%, +2.24%) |
|  |  | 8 | +0.75% (-14.43%, +7.46%) | -5.97% (-24.14%, +6.22%) | +1.49% (-15.67%, +10.45%) | -11.19% (-35.07%, +2.24%) |
|  |  | 9 | 0.0% (-17.16%, +7.21%) | -7.21% (-25.63%, +6.72%) | +0.75% (-16.44%, +9.7%) | -13.43% (-37.31%, +2.99%) |

#

# Table S5: Similarity between the model prediction and the original treatment decision on training and testing data, without radiomics and with radiomics, and for different model outcomes (OS and OS+DP).

In brackets are the 95% confidence intervals. Best results are shown in bold.

| *Mode Outcome* | *Radiomics* | *Hidden Layers* | *Training* | | | | *Testing* | | | |
| --- | --- | --- | --- | --- | --- | --- | --- | --- | --- | --- |
|  |  |  | *Decision 1 (Induction Chemo) Y/N* | *Decision 2 (CC / RT alone)* | *Decision 3 Neck Dissection (Y/N)* | *Overall* | *Decision 1 (Induction Chemo) Y/N* | *Decision 2 (CC / RT alone)* | *Decision 3 Neck Dissection (Y/N)* | *Overall* |
| Overall Survival | No | 0 | 53.23% (39.79%, 63.68%) | 51.99% (34.58%, 67.41%) | 53.98% (38.81%, 67.91%) | 52.65% (43.78%, 60.62%) | 53.73% (37.29%, 68.66%) | 52.24% (35.07%, 69.4%) | 53.73% (34.33%, 70.15%) | 52.99% (42.52%, 61.95%) |
|  |  | 1 | 52.74% (39.79%, 63.68%) | 52.24% (35.81%, 66.67%) | 71.64% (55.47%, 78.11%) | 58.37% (50.58%, 65.34%) | 53.73% (38.06%, 67.18%) | 51.49% (35.8%, 67.16%) | 71.64% (52.99%, 79.1%) | 58.46% (49.0%, 66.42%) |
|  |  | 2 | 55.47% (42.53%, 64.93%) | 56.72% (37.81%, 70.9%) | 67.16% (47.01%, 77.61%) | 59.37% (49.59%, 66.92%) | 56.72% (39.55%, 68.66%) | 55.97% (38.04%, 70.9%) | 66.42% (44.76%, 77.63%) | 58.96% (48.5%, 67.66%) |
|  |  | 3 | 56.72% (43.28%, 65.17%) | 59.95% (41.29%, 73.88%) | 77.61% (61.44%, 79.35%) | 64.1% (55.31%, 70.07%) | 57.46% (40.3%, 68.68%) | 58.96% (40.3%, 71.64%) | 77.61% (61.19%, **80.6%**) | 63.68% (54.73%, 70.9%) |
|  |  | 4 | 58.96% (39.3%, 65.17%) | 74.88% (46.27%, **78.11%**) | 78.61% (63.18%, **79.6%**) | 69.49% (59.12%, 73.47%) | 61.19% (36.57%, 69.4%) | 70.9% (47.76%, 73.88%) | 79.1% (62.67%, 79.85%) | 68.91% (57.71%, 73.38%) |
|  |  | 5 | 56.72% (38.56%, 64.68%) | 74.38% (39.28%, **78.11%**) | 77.11% (47.75%, **79.6%**) | 67.33% (53.56%, 72.97%) | 58.21% (35.82%, 69.4%) | 70.15% (41.79%, 73.88%) | 77.61% (47.0%, 79.85%) | 66.42% (52.72%, 73.13%) |
|  |  | 6 | 55.47% (37.56%, 64.43%) | 70.15% (30.09%, 77.86%) | 72.39% (34.33%, 79.1%) | 63.27% (46.01%, 71.64%) | 55.22% (32.84%, 68.68%) | 67.16% (32.84%, 73.88%) | 72.39% (33.58%, 79.85%) | 62.19% (45.77%, 71.89%) |
|  |  | 7 | 52.99% (37.31%, 63.68%) | 65.05% (26.87%, 77.36%) | 67.16% (28.6%, 78.86%) | 59.08% (42.04%, 70.15%) | 52.99% (33.56%, 68.66%) | 62.69% (29.85%, 73.13%) | 67.16% (28.36%, 79.85%) | 58.46% (41.78%, 70.15%) |
|  |  | 8 | 51.74% (37.31%, 63.43%) | 59.58% (25.12%, 77.11%) | 61.94% (25.86%, 78.61%) | 55.64% (38.56%, 68.58%) | 51.49% (32.82%, 68.66%) | 58.21% (28.36%, 73.13%) | 61.19% (25.37%, 79.85%) | 55.47% (38.31%, 68.66%) |
|  | Yes | 0 | 52.74% (40.3%, 63.68%) | 51.24% (34.58%, 66.67%) | 53.86% (38.56%, 68.16%) | 52.32% (43.94%, 61.12%) | 52.99% (35.82%, 67.91%) | 50.75% (33.56%, 67.91%) | 53.73% (35.07%, 69.4%) | 52.49% (42.29%, 62.69%) |
|  |  | 1 | 53.98% (41.54%, 63.93%) | 54.98% (37.81%, 69.65%) | 71.89% (55.22%, 78.36%) | 59.78% (51.82%, 66.67%) | 54.48% (38.06%, 67.18%) | 54.48% (37.31%, 68.66%) | 71.64% (52.22%, 79.1%) | 59.7% (50.5%, 67.41%) |
|  |  | 2 | 57.46% (44.53%, 65.42%) | 62.19% (44.03%, 73.39%) | 76.62% (65.92%, 79.35%) | 64.68% (57.63%, 70.15%) | 58.96% (41.79%, 69.4%) | 60.45% (41.79%, 72.39%) | 76.12% (64.18%, **80.6%**) | 64.43% (56.22%, 70.65%) |
|  |  | 3 | 58.71% (43.77%, 65.67%) | 71.39% (53.23%, 77.62%) | 78.36% (69.65%, **79.6%**) | 68.82% (61.44%, 72.8%) | 60.45% (41.79%, **70.15%**) | 67.91% (51.49%, **74.63%**) | 78.36% (68.66%, 79.85%) | 68.16% (59.7%, 73.13%) |
|  |  | 4 | 58.58% (40.05%, 65.17%) | 74.38% (50.49%, 77.86%) | 78.86% (63.17%, **79.6%**) | 69.15% (59.7%, 73.22%) | 60.45% (35.82%, 69.4%) | 70.15% (50.75%, **74.63%**) | 79.1% (64.14%, **80.6%**) | 68.91% (59.45%, 73.39%) |
|  |  | 5 | 57.21% (38.31%, 64.43%) | 74.38% (37.55%, 77.86%) | 77.36% (50.24%, 79.35%) | 67.21% (54.3%, 72.72%) | 58.96% (34.31%, 69.4%) | 70.15% (41.03%, 73.88%) | 77.61% (48.51%, 79.85%) | 66.67% (52.98%, 72.89%) |
|  |  | 6 | 54.23% (38.06%, 64.43%) | 70.15% (29.1%, 77.86%) | 73.38% (33.83%, 79.1%) | 63.43% (47.68%, 71.73%) | 55.97% (34.31%, 68.66%) | 66.42% (31.34%, 73.88%) | 73.88% (33.58%, 79.85%) | 62.44% (46.51%, 71.64%) |
|  |  | 7 | 52.61% (37.31%, 63.43%) | 63.93% (25.62%, 77.36%) | 68.91% (28.6%, 79.1%) | 59.25% (42.54%, 69.82%) | 52.61% (32.82%, 67.91%) | 61.94% (29.83%, 73.13%) | 68.66% (29.09%, 79.85%) | 58.46% (41.79%, 70.15%) |
|  |  | 8 | 51.99% (37.56%, 63.68%) | 59.33% (24.62%, 77.11%) | 61.19% (23.38%, 78.61%) | 55.64% (37.89%, 68.99%) | 51.49% (32.84%, 68.66%) | 57.46% (28.36%, 73.13%) | 61.94% (23.13%, 79.12%) | 55.22% (38.3%, 69.4%) |
|  |  | 9 | 51.99% (37.56%, 63.18%) | 54.98% (24.63%, 77.11%) | 59.2% (24.63%, 78.36%) | 53.98% (36.48%, 68.33%) | 52.24% (32.84%, 68.66%) | 53.73% (28.36%, 73.13%) | 59.7% (24.63%, 79.1%) | 53.98% (36.82%, 68.91%) |
| Overall Survival and Dysphagia | No | 0 | 53.98% (39.55%, 63.93%) | 51.0% (34.58%, 66.92%) | 71.39% (53.98%, 77.86%) | 58.46% (49.59%, 66.17%) | 55.22% (37.31%, 68.66%) | 51.49% (32.84%, 68.66%) | 71.64% (51.47%, 79.1%) | 58.46% (49.0%, 67.41%) |
|  |  | 1 | 55.47% (42.79%, 65.42%) | 66.17% (48.25%, 75.62%) | 77.86% (67.91%, 79.35%) | 65.88% (58.87%, 70.98%) | 56.72% (38.79%, 69.4%) | 63.43% (45.52%, **74.63%**) | 77.61% (67.16%, **80.6%**) | 65.17% (57.46%, 71.65%) |
|  |  | 2 | 57.71% (45.27%, **66.42%**) | 75.37% (64.92%, **78.11%**) | **79.1%** (76.11%, 79.35%) | 70.4% (65.34%, 73.63%) | 58.96% (42.52%, 68.66%) | 70.9% (60.45%, **74.63%**) | **79.85%** (75.37%, 79.85%) | 69.65% (63.43%, 73.38%) |
|  |  | 3 | 59.2% (**47.26%**, 66.17%) | **77.11%** (**68.89%**, **78.11%**) | **79.1%** (**77.36%**, 79.35%) | **71.52%** (**66.5%**, **74.13%**) | 61.19% (**45.5%**, **70.15%**) | **72.39%** (**64.93%**, 73.88%) | **79.85%** (**77.61%**, 79.85%) | **70.65%** (**64.68%**, **73.88%**) |
|  |  | 4 | **59.95%** (41.29%, 65.67%) | 75.87% (58.7%, **78.11%**) | **79.1%** (**77.36%**, 79.35%) | 71.06% (63.68%, 73.63%) | **61.94%** (37.31%, **70.15%**) | 71.64% (57.44%, **74.63%**) | **79.85%** (76.87%, 79.85%) | 70.4% (62.19%, **73.88%**) |
|  |  | 5 | 57.71% (39.3%, 65.17%) | 76.12% (50.23%, **78.11%**) | 78.86% (70.88%, 79.35%) | 69.73% (59.62%, 73.55%) | 59.7% (34.33%, 69.4%) | 71.64% (50.73%, 73.88%) | **79.85%** (67.16%, 79.85%) | 69.15% (59.2%, 73.63%) |
|  |  | 6 | 56.22% (38.8%, 64.18%) | 72.89% (34.08%, 77.86%) | 77.36% (48.01%, 79.35%) | 66.5% (51.82%, 72.64%) | 58.21% (34.33%, 69.4%) | 68.66% (35.07%, **74.63%**) | 77.61% (47.76%, 79.87%) | 65.67% (50.75%, 72.89%) |
|  |  | 7 | 53.73% (37.56%, 63.93%) | 67.41% (29.35%, 77.86%) | 73.63% (34.81%, 79.1%) | 62.56% (46.43%, 71.23%) | 54.48% (33.58%, 68.66%) | 64.18% (31.32%, 73.13%) | 73.88% (34.31%, 79.85%) | 61.69% (45.52%, 71.39%) |
|  |  | 8 | 52.24% (37.56%, 63.69%) | 61.69% (26.12%, 77.11%) | 66.42% (28.6%, 78.86%) | 57.63% (41.29%, 69.49%) | 52.99% (32.84%, 67.91%) | 60.45% (29.1%, 73.13%) | 65.67% (26.87%, 79.85%) | 57.21% (41.04%, 69.65%) |
|  | Yes | 0 | 52.99% (40.05%, 64.18%) | 51.49% (35.32%, 66.92%) | 53.73% (38.05%, 67.16%) | 52.57% (44.28%, 60.7%) | 53.73% (35.82%, 67.91%) | 51.49% (34.33%, 67.91%) | 53.73% (35.82%, 68.66%) | 52.24% (42.54%, 61.7%) |
|  |  | 1 | 53.98% (42.03%, 63.94%) | 55.72% (38.31%, 69.65%) | 71.89% (56.72%, 78.36%) | 59.87% (52.16%, 66.67%) | 54.48% (39.53%, 67.16%) | 54.48% (37.31%, 69.4%) | 71.64% (54.48%, 79.1%) | 59.7% (51.24%, 67.41%) |
|  |  | 2 | 54.23% (42.54%, 64.43%) | 53.36% (36.56%, 69.65%) | 76.37% (64.66%, 79.35%) | 60.95% (53.65%, 68.0%) | 54.48% (39.55%, 67.91%) | 52.99% (35.07%, 68.66%) | 76.12% (62.69%, **80.6%**) | 60.7% (52.24%, 68.41%) |
|  |  | 3 | 56.22% (43.53%, 65.17%) | 62.44% (43.77%, 74.88%) | 78.86% (70.89%, 79.35%) | 65.34% (58.37%, 70.48%) | 56.72% (40.3%, 68.66%) | 59.7% (41.79%, 72.39%) | **79.85%** (70.88%, **80.6%**) | 64.93% (57.46%, 71.14%) |
|  |  | 4 | 59.2% (40.29%, 65.17%) | 75.62% (55.21%, **78.11%**) | 78.86% (62.44%, 79.36%) | 70.07% (59.95%, 73.38%) | **61.94%** (36.57%, 69.42%) | 70.9% (52.97%, 73.88%) | 79.1% (61.18%, 79.85%) | 69.4% (58.71%, 73.64%) |
|  |  | 5 | 57.21% (38.06%, 64.43%) | 74.38% (36.07%, **78.11%**) | 77.61% (45.01%, 79.35%) | 67.0% (52.9%, 72.89%) | 59.7% (34.33%, 69.42%) | 70.15% (38.06%, 73.88%) | 77.61% (44.03%, 79.85%) | 66.17% (52.73%, 73.13%) |
|  |  | 6 | 54.48% (38.06%, 64.18%) | 69.65% (28.59%, 77.86%) | 73.13% (33.33%, 79.1%) | 63.39% (46.68%, 71.89%) | 55.97% (33.58%, 68.66%) | 66.42% (32.09%, 73.88%) | 73.13% (32.09%, 79.85%) | 62.69% (46.27%, 71.89%) |
|  |  | 7 | 52.49% (37.31%, 63.18%) | 63.93% (26.11%, 77.12%) | 67.04% (30.09%, 78.86%) | 58.62% (40.54%, 69.74%) | 52.99% (32.09%, 68.66%) | 61.94% (29.85%, 73.13%) | 67.16% (26.87%, 79.85%) | 58.21% (40.3%, 69.66%) |
|  |  | 8 | 51.62% (37.81%, 63.43%) | 58.08% (25.61%, 77.11%) | 61.19% (24.87%, 78.61%) | 55.56% (38.56%, 68.41%) | 52.24% (32.84%, 68.66%) | 56.72% (28.36%, 73.13%) | 61.19% (24.63%, 79.1%) | 54.98% (37.81%, 68.41%) |
|  |  | 9 | 51.0% (37.56%, 63.43%) | 56.22% (24.87%, 77.11%) | 57.09% (24.87%, 78.36%) | 53.32% (37.98%, 67.74%) | 51.49% (32.09%, 68.66%) | 55.22% (28.36%, 72.39%) | 56.72% (23.88%, 79.1%) | 53.23% (37.56%, 67.66%) |
